# Donor characteristics and their impact on kidney transplantation outcomes: Results from two nationwide instrumental variable analyses based on outcomes of donor kidney pairs accepted for transplantation

**DOI:** 10.1016/j.eclinm.2022.101516

**Published:** 2022-06-25

**Authors:** Alexander F. Schaapherder, Maria Kaisar, Lisa Mumford, Matthew Robb, Rachel Johnson, Michèle J.C. de Kok, Frederike J. Bemelman, Jacqueline van de Wetering, Arjan D. van Zuilen, Maarten H.L. Christiaans, Marije C. Baas, Azam S. Nurmohamed, Stefan P. Berger, Esther Bastiaannet, Aiko P.J. de Vries, Edward Sharples, Rutger J. Ploeg, Jan H.N. Lindeman

**Affiliations:** a Department of Surgery and Leiden Transplant Center, Leiden University Medical Center, Leiden, the Netherlands; b Nuffield Department of Surgical Sciences, University of Oxford, Oxford, United Kingdom; c Research and Development, NHS Blood and Transplant, Bristol & Oxford, United Kingdom; d Statistics and Clinical Studies, NHS Blood and Transplant, Bristol, United Kingdom; e Department of Internal Medicine (Nephrology), Amsterdam UMC, Academic Medical Center, Amsterdam, the Netherlands; f Department of Internal Medicine (Nephrology), Erasmus University Medical Center, Rotterdam, the Netherlands; g Department of Internal Medicine (Nephrology), University Medical Center Utrecht, Utrecht, the Netherlands; h Department of Internal Medicine (Nephrology), Maastricht University Medical Center, Maastricht, the Netherlands; i Department of Internal Medicine (Nephrology), Radboud University Medical Center, Nijmegen, the Netherlands; j Department of Internal Medicine (Nephrology), Amsterdam UMC, VU Medical Center, Amsterdam, the Netherlands; k Department of Internal Medicine (Nephrology), University Medical Center Groningen, Groningen, the Netherlands; l Department of Surgery, Leiden University Medical Center, Leiden, The Netherlands. Current address: Dept. Epidemiology, UZH, Zurich, Switzerland; m Division of Nephrology, Department of Internal Medicine and Leiden Transplant Center, Leiden University Medical Center, Leiden, the Netherlands

**Keywords:** Kidney transplantation, Outcomes, Risk, Prediction algorithm, Instrumental variable analysis, Donor characteristics

## Abstract

**Background:**

Donor-characteristics and donor characteristics-based decision algorithms are being progressively used in the decision process whether or not to accept an available donor kidney graft for transplantation. While this may improve outcomes, the performance characteristics of the algorithms remains moderate. To estimate the impact of donor factors of grafts accepted for transplantation on transplant outcomes, and to test whether implementation of donor-characteristics-based algorithms in clinical decision-making is justified, we applied an instrumental variable analysis to outcomes for kidney donor pairs transplanted in different individuals.

**Methods:**

This analysis used (dis)congruent outcomes of kidney donor pairs as an instrument and was based on national transplantation registry data for all donor kidney pairs transplanted in separate individuals in the Netherlands (1990-2018, 2,845 donor pairs), and the United Kingdom (UK, 2000-2018, 11,450 pairs). Incident early graft loss (EGL) was used as the primary discriminatory factor. It was reasoned that a scenario with a dominant impact of donor variables on transplantation outcomes would result in high concordance of EGL in both recipients, whilst dominance of asymmetrical outcomes could indicate a more complex scenario, involving an interaction of donor, procedural and recipient factors.

**Findings:**

Incidences of congruent EGL (Netherlands: 1·2%, UK: 0·7%) were slightly lower than the arithmetical (stochastic) incidences, suggesting that once a graft has been accepted for transplantation, donor factors minimally contribute to incident EGL. A long-term impact of donor factors was explored by comparing outcomes for functional grafts from donor pairs with asymmetrical vs. symmetrical outcomes. Recipient survival was similar for both groups, but a slightly compromised graft survival was observed for grafts with asymmetrical outcomes in the UK cohort: (10-years Hazard Ratio for graft loss: 1·18 [1·03-1·35] p<0·018); and 5 years eGFR (48·6 [48·3–49·0] vs. 46·0 [44·5–47·6] ml/min in the symmetrical outcome group, p<0·001).

**Interpretation:**

Our results suggest that donor factors for kidney grafts deemed acceptable for transplantation impact minimally on transplantation outcomes. A strong reliance on donor factors and/or donor-characteristics-based decision algorithms could result in unjustified rejection of grafts. Future efforts to optimize transplant outcomes should focus on a better understanding of the recipient factors underlying transplant outcomes.

**Funding:**

None.

**Research in context**

**Evidence before this study**

Donor-characteristics, and donor characteristics-based algorithms are often used in the decision-process whether or not to accept an offered donor kidney for transplantation. Yet, epidemiological evaluations suggests that donor factors and decision-algorithms (risk indices) only modestly predict outcomes and therefore a reliance only on the donor factors and/or decision algorithms may result in discard of potentially suitable grafts.

**Added value of this study**

Using national registry data for the Netherlands and the UK, an instrumental variable analysis based on an outcome comparison of donor kidney pairs, that were transplanted in two different recipients, was applied. Results from this evaluation suggest that once kidneys are considered eligible for transplantation, donor factors minimally impact outcomes.

**Implications of all available evidence**

A strong focus on donor factors in the decision-making process whether or not to accept an offered graft for transplantation might result in unjustified rejection or even discard of viable donor organs.

Alt-text: Unlabelled box

## Introduction

In an era of pressing donor shortages, decisions when to accept or decline a donor graft for transplantation, and questions how to optimize allocation of available donor organs remain major challenges to the field.^1^^,^^2^ With the aim of guiding clinicians in the decision-making process, several supporting algorithms have been formulated. In particular the Kidney and Liver Donor Risk Indices, and their derivatives have now been widely implemented in clinical practice.^3^^,^^4^ Yet, despite their broad acceptance, reports consistently conclude that the performance of the algorithms is moderate at max.^5^, ^6^, ^7^

The modest performance of donor risk indices may reflect incomplete attribution of relevant donor aspects, failure to fully capture all procedural characteristics in the donation and procurement process and/or a possible recipient-donor organ interaction. Moreover, since most risk indices relate to 5-year post-transplant outcomes, they are affected by differences in the quality and organization of post-transplant care.^8^ A final, critical point is that the risk indices are devised as allocation/procedural quality assessment tools.^9^ As such, they typically do not include recipient characteristics and consequently, the likely impact of recipient-associated factors on the transplantation outcome is ignored, and remains in fact unknown. An alternative, and none exclusive explanation is that -once donor grafts have been accepted for transplantation- donor factors have a limited impact on transplantation outcomes.

In an effort to estimate the contribution of donor factors (of grafts accepted for transplantation) to transplantation outcomes in kidney transplantation, we here report an instrumental variable analysis^10^ based on an outcome comparison of kidney pairs as an instrument. To be more specific we compared the outcomes for grafts from a single donor that were donated to, and transplanted into separate recipients. The primary focus of the study was on early graft loss (EGL, i.e. all graft losses occurring within 90-days of transplantation) as this represents the most unambiguous short-term outcome measure. In addition, compared to other outcomes such as 5-years eGFR and/or graft survival, EGL is less affected by differences in post-transplant care, as well as by recipient factors, such as recurrent renal disease.

It was reasoned, that a scenario with a dominant impact of donor variables on transplantation outcomes would result in high concordance of EGL in both recipients, whilst dominance of asymmetrical outcomes could indicate a more complex scenario, involving an interaction of donor, procedural and in particular recipient factors.

## Methods

The study is based on data from two national registries in The Netherlands and the United Kingdom (UK): the NOTR (Netherlands Organ Transplant Registry) and NTxD (UK National Transplant Database). The study is based on fully anonymized registry data, as such no formal medical ethical consent is required according to Dutch law. The scientific review boards of the NOTR and NTxD approved the study. The original first data-set retrieved from the NOTR, was analyzed and evaluated. To study the robustness of the findings, subsequently, a second similar data-set was generated in NTxD with help of the Clinical Trials and Studies Unit of NHS Blood and Transplant (NHSBT) in the UK for comparison with the results from the NOTR and to allow evaluation of long-term (i.e. day 90 and beyond) outcomes. Data used in the study is accessible through NOTR and NTxD.

The study protocol was approved by the respective governing committees of both NOTR that is hosted by the Dutch Transplant Foundation (NTS) and of UK NTxD that is hosted by the NHSBT. The reported clinical and research activities are consistent with the Principles of the Declaration of Istanbul as outlined in the 'Declaration of Istanbul on Organ Trafficking and Transplant Tourism. Patients and public were not involved in the study.

The NOTR and NTxD are both mandatory nationwide registries that include granular donor and recipient demographics, procedural and outcome data of all types of organ transplantation carried out in both The Netherlands and the UK, and include data provided by the organ procurement organization as well as all participating transplant centers. In the first year after transplantation, registry follow-up is at month 3, thereafter on a yearly basis. Quality checks are performed by on-site polls, business rules in application and cross checks with the national dialysis registry. Data on multiple variables as kidney donor and recipient characteristics as well as transplant outcomes for all procedures performed in The Netherlands from 1st January 1990 through 1st January 2018, and subsequently in the UK from 1st January 2000 through 1st January 2018 were retrieved. There were no missing data for type of donor, graft survival or 90-day recipient survival in both data sets. Sixteen respectively 27% of the 1 and 5 years eGFRs values used for the secondary outcome analysis were missing. Missing values were handled as -missing at random- and list-wise deleted.

This study only includes data for procedures in which both donor kidneys were transplanted in two different recipients and for which outcome information for both kidneys was available. Combined organ procedures including paired kidney transplantations, procedures involving recipients younger than 18 years old, and procedures with grafts donated after uncontrolled circulatory death (i.e. Maastricht Category I: dead on arrival and II: unsuccessful resuscitation) were excluded from the analysis.

### Definitions

EGL was defined as graft loss within 90 days after transplantation. Delayed Graft Function (DGF) was defined as the need for dialysis in the first postoperative week(s). The Modification of Diet in Renal Disease (MDRD) equation was used to estimate the glomerular filtration rate (eGFR). The non-scaled, donor-only version of the Kidney Donor Risk Index (KDRI) was calculated as described by Rao et al.^12^ The following definitions were used for ischemic periods of the donor kidneys. The first warm ischemic period is the time following the no touch period after circulatory arrest and asystole in the DCD donor, until cold flush-out in the donor is commenced. The cold ischemic period is the time from start of cold flush-out until the start of the vascular anastomosis in the recipient. The graft anastomosis time is defined as the time from kidney removal from static cold storage or hypothermic machine perfusion until reperfusion in the recipient.

Main (primary) outcomes for the study were: (I) the observed vs. predicted concordance of EGL for donor kidney pairs, and (II) long-term graft outcomes (i.e. recipient and graft survival; 1 and 5-years eGFR) of functional kidneys -from donor pairs from which the contralateral graft experienced EGL- vs. symmetrically functional pairs (no EGL). Main exposure variables are summarized in Tables 1 and 2.

### Data analysis

IBM SPSS Statistics 23.0 (IBM Corp., Armonk, NY, USA) was used for statistical analysis. Comparisons between the groups were performed using the independent t-test for normal-distributed data, the Mann-Whitney rank test for non-parametric data, and the chi-square test for categorical data.

Simple univariate correlations were performed to map correlations between donor, procurement and recipient characteristics and outcomes. Recipient and graft survival were depicted with Kaplan-Meier curves, and survival differences explored through log-rank tests. Cox proportional hazards models were applied to estimate differences in the risk of recipient mortality or in graft loss. All survival models were adjusted (Cox-proportional hazards model) to calculate the Hazard Ratio (HR) for variables statistically relevant (p-value <0.10) in the univariate analysis. Results are represented as HR with the corresponding 95% confidence intervals (CI). (Backward) binary logistic regression analyses, and Analysis of Covariance (ANCOVA) were used to examine the association between donor, procedural and recipient characteristics, and functional outcomes (incident EGL, and 1 and 5-year eGFR). Factors included in the binary logistic regression analyses and covariates included in the ANCOVA are summarized in the table legends.

The amount of variation explained by the variable or set of variables was estimated by r^2^ for the simple correlations or Nagelkerke's r^2^ for the regression analyses.

Missing data was considered as missing at random. There was less than 2% missing data for the primary outcome graft survival and key characteristics such as donor and recipient age and sex, donor type and cold ischemia time.

### Sensitivity analyses

The primary analysis was based on death-censored EGL, i.e. excluding patients who died within 90 days after transplantation with a functioning graft. The following three sensitivity analyses were performed: (1) group allocation based on a more extended definition of EGL that included all graft losses occurring in the first 90 days following transplantation (i.e. including graft loss caused by recipient death within the first 90 days) as the primary outcome measure, and (2) and (3) group allocation based on more restricted (more donor-factors focused) definitions i.e.: (2) only EGL attributed to primary non function, thrombosis or vascular operative problems, or (3) exclusively EGL as the consequence of primary non function.

### Role of the funding source

There were no specific funding sources involved in the study. Access to the data set was handled by CK and LM. The decision to submit for publication was unanimously made by all authors.

## Results

**Figure 1:**
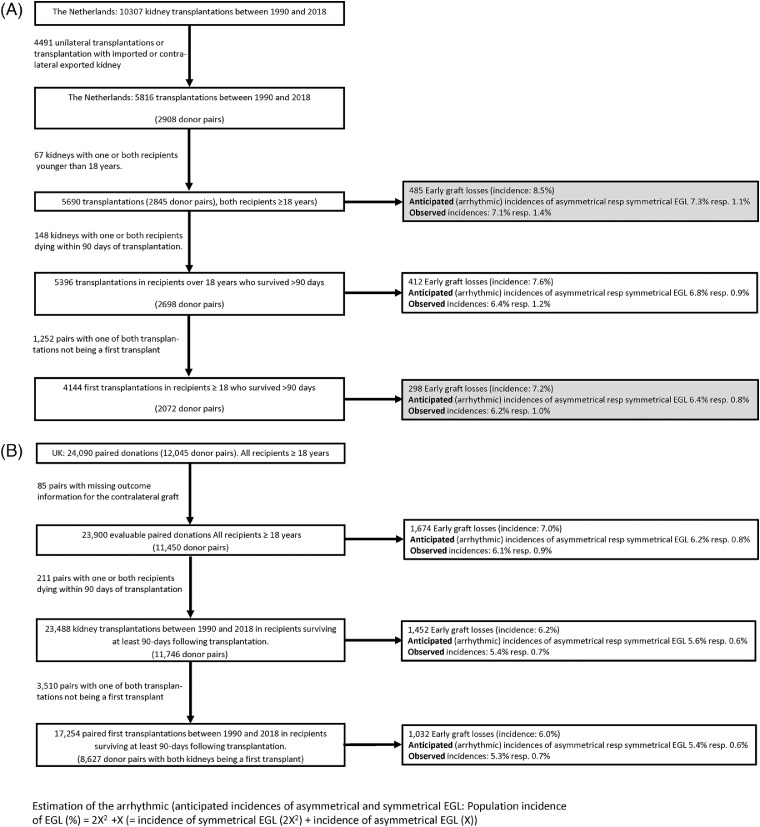
(A) Flow diagram of the cohort selection, and observed and anticipated (arrhythmic)* distribution of symmetrical (paired) and asymmetrical Early Graft Loss in The Netherlands. The primary analysis was performed for death-censored EGL (white box). Two sensitivity analysis were performed (shaded boxes): one based on all cases of EGL (not death-censored), and a second exclusively focusing on for primary transplantations. *) Anticipated (arrhythmic) distribution of symmetrical and asymmetrical EGL equals: observed incidence of EGL (%) = 2X^2^ + X (i.e. incidence of symmetrical EGL equals 2X^2^; incidence of asymmetrical EGL equals X). (B) Flow diagram of the cohort selection, and observed and anticipated (arrhythmic)* distribution of symmetrical (paired) and asymmetrical Early Graft Loss in the United Kingdom. The primary analysis was performed for death-censored EGL (white box). Two sensitivity analysis were performed (shaded boxes): one based on all cases of EGL (not death-censored), and a second exclusively focusing on for primary transplantations. *) Anticipated (arrhythmic) distribution of symmetrical and asymmetrical EGL equals: observed incidence of EGL (%) = 2X^2^ + X (i.e. incidence of symmetrical EGL equals 2X^2^; incidence of asymmetrical EGL equals X).

The impact of donor factors (of grafts accepted for transplantation) to transplantation outcomes in kidney transplantation was first explored by evaluating the concordance of EGL for transplanted donor pairs. It was reasoned that a high concordance would be consistent with prominent role of donor factors on outcome, and a low concordance would be consistent with a more modest role. A first exploration was performed for all paired transplantations performed in the 1990-2018 interval in the Netherlands. Out of the 10307 transplantations, paired outcome data was available for 5,816 kidneys (Figure 1A). For 4,491 procedures outcome data for the contralateral kidney was absent. Reasons for missing information on the contralateral graft were asymmetrical death of the recipient with a functioning graft within 90-days of transplantation, or because the kidney was imported, or the contralateral kidney was exported in the context of the Eurotransplant international kidney exchange program, or finally because the contralateral kidney was not transplanted. An additional 210 pairs were excluded because one or both kidneys were transplanted in a recipient younger dan 18, or because one or both recipients died within 90 days of transplantation (Figure 1A). Donor, procedural and recipient characteristics of the 5,396 paired transplantations are summarized in Tables 1A and 1B. Statistically significant, but clinically weak correlations were observed between less favorable donor, procedural and recipient profiles, and incident EGL (Table 2).

**Table 1A:** *Donor characteristics of 5396 Dutch paired transplantations performed between 1990 and 2018. (EGL censored for death with functioning graft).* Mean (sd) or Median [IQR].

	Double sided EGL n=66 33 donor pairs	Asymmetrical EGL n=692 346 donor pairs	Symmetrical no EGL “Reference” n=4638 2319 donor pairs
% DCD	51.5	46.5	44.1

Sex donor (% male)	66.7	52.3	53.5

Age donor (yr) mean (sd) median [IQR]	51.4 (15.2) 54.0 [40.8-64.3]	52.6 (14.1) 54.0 [44.8 - 61.3]	48.9 (15.7) 52.0 [40-61.3]

Body-mass index donor median [IQR]	26.9 (5.8) 24.7 [23.0 – 29.8]	25.6 (5.0) 24.7 [22.5 – 27.7]	24.9 (4.1) 24.5 [22.5 – 26.8

% Donors >60 yrs	36.7	29.8	23.8

% Expanded Criteria Donor#	39.4	36.4	32.8

Last creatinine donor (μmol/l) median [IQR]	88.1 (33.0) 85.0 [67.8 – 105.3]]	80.0 (37.8) 70.7 [56.2 - 91.0]	77.5 (36.7) 71.0 [56.0 – 89.0]

eGFR (MDRD) donor (ml/min) median [IQR]	89.0 (33.0) 84.1 [67.5 – 107.1]	97.5 (37.6) 92.8 [71.0 – 116.0]	102.5 (40.7) 96.7 [75.3 – 122.5]

Cause of death donor (%) Trauma Stroke Cardiac arrest Other	21.2 66.7 0.0 12.1	20.8 58.7 6.9 13.6	28.0 49.8 7.8 14.4

Hypertension donor (%) No Yes Unknown	63.6 21.2 15.2	57.2 23.4 19.4	63.3 20.4 16.3

Diabetes donor (%) No Yes Unknown	39.4 9.1 51.5	54.3 6.4 39.3	63.6 3.6 32.8

Smoking donor (%) No Yes Unknown	33.3 42.4 24.3	34.9 43.4 21.7	41.0 42.1 16.9

# Expanded criteria donor: over 50 years of age with 2 or more of the following conditions: history of hypertension, serum creatinine ≥ 133 µmol/L or cause of death from stroke. ^†^Starting 2016 all grafts were machine perfused.

**Table 1B:** *Procedural and recipient characteristics of 5396 Dutch paired transplantations performed between 1990 and 2018. (EGL censored for death with functioning graft).* Mean (sd) or Median [IQR].

	Double sided EGL n=66	Asymmetrical EGL n=346	Asymmetrical functioning graft n=346	Symmetrical no EGL “Reference” n=4638
Left kidney (%)	50	45.1	54.9	50

First warm ischemia time (min) (DCD only) Mean (sd) Median IQR	24.4 (9.2) 20.0 [17.0-31.5]	21.1 (9.8) 20.0 [16.0-26.0]	21.5 (9.5) 20.0 [16.0-26.0]	17.9 (7.1) 17.0 [14.0-21.0]

Cold ischemia time (hrs) Cold ischemia time distribution (%) <12 hrs 12-18 hrs 18-24 hrs >24 hrs Unknown	22.7 (8.4) 12.1 16.7 27.3 40.9 3.0	21.0 (7.4) 10.1 25.4 31.5 26.0 6.9	20.4 (7.4) 11.8 28.0 29.5 25.1 5.5	18.8 (7.4) 16.6 32.8 25.3 18.9 6.5

Graft anastomosis time (min) Median {IQR]	38.4 (16.5) 35.0 [28.5-45.0]	39.1 (16.5) 36.0 [29.0-45.0]	34.4 (13.1) 32.5 [26.0-40.0]	34.2 (13.1) 32.0 [25.0-40.0]

% DGF	NA	NA	46.4	39.0

Sex recipient (% male)	68.2	58.4	61.8	61.8

Age recipient (years)	51.9 (14.6) 54.5 [40.0-64.3]	52.0 (14.0) 55.0 [41.0-63.0]	51.8 (13.0) 53.0 [42.0-62.0]	52.9 (13.4) 57.0 [35.0-70.0]

BMI recipient (kg/m²)	26.5 (4.8)	26.2 (4.6)	25.5 (4.3)	25.5 (4.4)

% First transplant	77.3	82.7	87.9	87.4

Years on dialysis	4.1 (1.7)	4.1 (2.5)	3.9 (2.0)	3.9 (2.0)

Mismatches (%) HLA-Dr 0 1 2 HLA-A 0 1 2 HLA-B 0 1 2	37.9 59.1 3.0 30.3 56.1 13.6 28.8 51.5 19.7	33.0 58.8 8.1 27.0 54.2 18.8 11.9 74.5 18.8	34.8 59.4 5.8 32.5 50.5 17.1 13.6 67.0 19.4	36.3 56.1 7.6 27.5 51.3 16.2 17.1 57.6 25.3

% of recipients with panel reactive antibodies >6%	19.7	17.1	9.2	11.1

Cause of early graft loss (%) Rejection Prim. non function Thrombosis/infarction Technical Infection Recurrent disease Other	19.7 40.9 19.7 10.6 0.0 1.5 7.6	25.7 27.2 24.6 15.3 3.0 2.6 3.8

**Table 2:** Simple, univariate correlations (Spearman's correlation coefficients) between donor, procedural and recipient characteristics, and incident EGL (death censored).

	Dutch Cohort		UK Cohort	
	r	p	r	p
Year of transplant	-0.042	0.001	0.030	0.001
Donor age (years)	0.048	0.0001	0.053	0.0001
Donor sex (0=male; 1=female)	0.08	0.545	-0.003	0.654
Donor BMI (kg/m^2^)	-0.009	0.513	0.015	0.025
Donor cause of death (0=Trauma; 1: Cerebral Vascular Accident; 2: Circulatory Accident)	0.005	0.724	0.027	0.001
Donor history of CVD (no is reference)	DNA	DNA	0.025	0.0001
Donor hypertension (0=no; 1=yes)	0.009	0.533	0.032	0.0001
Donor diabetes (0=no; 1=yes)	-0.042	0.010	0.009	0.160
Donor smoking (0=no; 1=yes)	0.000	0.986	0.008	0.198
Cold ischemia time (hrs.)	0.028	0.040	0.022	0.001
Anastomosis time (min.)	0.012	0.375	0.022	0.180
Type cadaveric (0=DBD; 1=DCD)	0.008	0.518	0.007	0.314
Recipient age (years)	0.038	0.04	0.023	0.0001
Recipient sex (0=no; 1=yes)	0.015	0.250	0.003	0.695
Recipient BMI (kg/m^2^)	0.003	0.830	0.042	0.0001
Recipient DM (0=no; 1=yes)	DNA	DNA	0.011	0.096
Highly sensitized recipient (0=no; 1=yes)	-0.02	0.878	0.019	0.004
Recipient mismatch A (0=no; 1= 1 mismatch, 2= 2 mismatches)	0.001	0.935	0.012	0.067
Recipient mismatch B (0=no; 1= 1 mismatch, 2= 2 mismatches)	0.024	0.071	0.013	0.044
Recipient mismatch DR (0=no; 1= 1 mismatch, 2= 2 mismatches)	0.014	0.274	0.031	0.001

“type cadaveric is included as a variable, consequently first warm ischemia time (absent in DBD) is not included in the analysis). DNA: data not available.

The overall EGL_death censored_ rate in the Dutch cohort of 5,396 kidneys was 7·6%, with an incidence of double-sided (symmetrical) EGL of 1·2% (Figure 1A). This latter percentage is slightly higher than the anticipated arithmetical (stochastic) incidence for double-sided EGL (0.9 %) (see Figure 1A for the estimation of the arithmetical distribution). Nearly 85% of the EGL's were found to be asymmetrical. Since early (within 90-days) death following transplantation may relate to poor graft function, and incident EGL following retransplantation may involve accumulation of recipient-related risk-factors,^11^ two sensitivity analysis were performed (Figure 1A). One for all procedures in recipients over 18, i.e. also including recipients dying within 90-days of transplantation. The second sensitivity analysis exclusively incorporated primary transplants. Conclusions from these sensitivity analyses were similar to the primary analysis (Figure 1A).

To allow a more detailed evaluation in a comparable, larger cohort, and to overcome potential confounding related to selection bias and missing information caused by the Eurotransplant exchange program (non-random import or export of the contralateral graft), national registry data (NTxD) for the UK were used for validation and further exploration. The distribution of different donor types such as donation after brain death (DBD), extended criteria donation (ECD) and controlled donation after circulatory death (DCD) is comparable in The Netherlands and the UK. However, as the UK does not participate in an international organ exchange program, interference caused by missing data related to cross-border exchange is not present. It was decided to limit the evaluation to the 2000-2018 interval in the UK in order to reduce the impact of time-related changes in incident EGL and outcomes.^12^ This interval concerned 24,090 (12,045 donor pairs) transplantations for which paired information was available (Figure 1B). Donor, procedural and recipient characteristics of the 24,090 paired transplantations are summarized in Tables 3A and 3B.

**Table 3A:** *Donor characteristics of 23488 paired transplantations performed in the UK between 2000 and 2018. (EGL censored for death with functioning graft).* Mean (sd) or Median [IQR].

	Double sided EGL n=176^1^ 88 donor pairs	Asymmetrical EGL n=2552 1276 donor pairs	Symmetrical no EGL “Reference” n=20760 10380 donor pairs
% DCD	43.2	30.7	31.5
Sex donor (male) %	65.9	52.0	53.6
Age donor (yrs) mean (sd) median [IQR]	56.3 (12.0) 58.5 [50-65]	52.6 (13.3) 54 [45-62]	49.9 (15.0) 52.0 [41.0-60.0]
% Donors >60 yrs	48.9	32.1	27.2
Body-mass index donor (kg/m^2^)	26.6 (6.3)	27.1 (5.3)	26.6 (5.2)
Last creatinine donor (μmol/l) median [IQR]	97 (64) 85 [61-116]	86 (45) 76 [61-99]	85 (51) 74 [58-96]
eGFR (MDRD) donor (ml/min)	80.5 [54.3 – 116.0]	84.1 [63.5 – 107.3]	85.9 [64.3-110.6]
Cause of death donor (%) Trauma Stroke Cardiac arrest Other	23.3 62.8 2.3 11.6	20.8 70.2 0.9 8.0	27.8 62.3 1.1 8.8
Expanded criteria donor (%)#	56.1	43.7	35.5
History of diabetes (%)	3.5	7.2	6.0
History of hypertension (%)	35.0	32.0	25.8
History of smoking (%)	46.4	53.5	51.1

# Expanded criteria donor: over 50 years of age with 2 or more of the following conditions: history of hypertension, serum creatinine ≥ 133 µmol/L or cause of death from stroke.

**Table 3B:** *Procedural and recipient characteristics of 23488 paired transplantations performed in the UK between 2000 and 2018. (EGL censored for death with functioning graft).* Mean (sd) or Median [IQR].

	Double sided EGL n=176	Asymmetrical EGL n=1276	Asymmetrical functioning graft n=1276	Symmetrical no EGL “Reference” n=20760
Left kidney (%)	50	45.1	54.9	50

First warm ischemia time (min) (DCD only) Mean (sd) Median IQR	23.6 (9.1) 19.0 [14.0-32.0]	26.1 (27.3) 18.0 [14.0-25.0]	23.9 (22.6) 18.0 [15.0- 24.3]	22.5 (20.4) 18.0 [15.0-23.0]

Cold ischemia time (hrs) Cold ischemia time distribution (%) <12 hrs 12-18 hrs 18-24 hrs >24 hrs	16.5 (6.4) 30.8 40.9 20.7 7.7	17.0 (6.3) 23.9 40.3 24.8 10.9	16.3 (6.1) 27.0 41.9 22.2 8.9	16.2 (5.9) 24.5 43.9 22.2 9.4

Graft anastomosis time (min) Mean (sd) Median IQR	11.9 (4.0) 12.0 [10.0-14.8]	18.8 (27.1) 13 [11.0-16.0]	16.2 (19.6) 13.0 [11.0 – 16.0]	15.5 (19.4) 13.0 [10.0 – 15.0]

KDRI	1.12 (0.38)	1.08 (0.25)	1.07 (0.25)	1.08 (0.37)

DGF (%)	NA	NA	34.5	27.9

Sex recipient (male) %	59.7	62.7	63.7	63.0

Age recipient (years) Mean (sd) median [IQR]	52.5 (11.6) 54 [44-61.8]	51.1 (13.2) 52 [42-61]	55.0 (13.2) 52.0 [41.0- 61.0]	50.2 (13.3) 51.0 [41.0 – 60.0]

BMI recipient (kg/m²)	27.7 (4.4)	27.2 (4.9)	26.6 (4.8)	26.5 (4.9)

Diabetes (%)	9.1	9.1	7.9	8.5

Waiting time (years) Median (IQR))	2.3 [1.2 – 4.1]	2.4 [1.1 – 4.1]	2.1 [0.9 - 3.8]	2.1 [0.9 - 3.7]

Previous transplants 0 1 2 more	83.5 14.2 1.7 0.6	81.3 14.9 3.8 0.2	86.5 11.9 1.5 0.1	85.2 12.4 2.1 0.3

mismatches (%) HLA-Dr 0 1 2 HLA-A 0 1 2 HLA-B 0 1 2	48.9 43.8 7.4 19.9 54.5 25.6 14.2 72.2 13.6	53.1 43.2 3.7 23.3 48.6 28.1 18.0 68.8 13.2	56.4 39.5 4.1 24.4 51.3 24.3 19.6 66.7 13.7	57.0 39.5 3.5 23.2 50.7 26.1 19.2 67.9 12.9

Highly immunized patient (%)	11.9	10.3	7.8	8.3

Induction therapy Anti-IL2r ATG	69.9 3.5	68.7 4.6	69.0 4.3	72.7 3.4

Initial immune suppression (%) Azothioprine Ciclosporin A Tacrolimus Mycophenolate Corticosteroids	14.8 24.1 72.3 64.2 87.9	15.3 23.1 72.0 68.3 84.9	16.8 21.4 77.2 65.9 85.7	15.9 19.7 78.6 68.7 84.4

Cause of early graft loss % Rejection Primary non function Thrombosis/infarction Technical Infection Recurrent disease Other Not coded	10.8 14.2 17.6 14.2 1.7 1.1 15.3 25.1	9.7 13.0 16.2 14.7 0.9 0.7 20.2 24.6		

12 months eGFR (mL/min)	n.a.	n.a.	44.7 (18.4) 43.0 [32.0 – 56.0]	49.9 (19.1) 48.0 [36.0 – 61.0]

The overall incidence of EGL_death censored_ for the UK cohort was 6.2%, and the incidence of double-sided (symmetrical) EGL was 0·7% (Figure 1B). A percentage nearly equal to the anticipated stochastic, arithmetical incidence of 0.6% for double-sided EGL, and it was concluded that the large majority of incident EGL cases (87%) were asymmetrical (Figure 1B). Conclusions for sensitivity analyses that either included all EGL cases (i.e. also including those associated with early (≤90 day) mortality), respectively exclusively included primary transplantations (Figure 1B),^11^ or that differentiated between conventional vs. expanded criteria donors (supplemental Figure 1) were similar to the primary analysis.

Clear statistical, but clinically weak associations were found between more unfavorable donor characteristics such as donor age, a history of hypertension and extended criteria donor, and incident EGL (Table 2). Yet, the estimated (Nagelkerke's) r^2^ of a multivariate model indicated that the included donor, procedural and recipient factors only predict 2·7% (5·3% in the Dutch data set) of the variation in EGL (Table 4).

**Table 4:** Multivariable logistic regression evaluating associations of donor, procedural and recipient characteristics with incident (yes/no) Early Graft Loss* as outcome measure.

	Dutch Cohort Cox&Schnell r^2^: 0.024 Nagelkerke r^2^: 0.058 n=5,096 (373 cases with EGL)		UK Cohort Cox&Schnell r^2^: 0.05 Nagelkerke r^2^: 0.014 n=22,356 (1356 cases with EGL)	
	OR (95% CI)	p	OR (95% CI)	p
Year of transplant	0.964 (0.943-0.984)	0.001		ns
Donor age (year)	1.014 (1.006-1.023)	0.001	1.013 (1.009-1.018)	0.0001
Donor BMI (kg/m²)	1.032 (1.008-1.057)	0.009		ns
Donor hypertension (no is reference)	>20% Missing		0.880 (0.776-0.997)	0.044
Cold ischemia time (hours)	1.030 (1.013-1.048)	0.001	1.027 (1.018-1.035)	0.0001
Anastomosis time (minutes)	1.019 (1.013-1.026)	0.0001	>20% Missing	
Type cadaveric (DBD is reference)	1.775 (1.387-2.273)	0.0001		ns
Recipient Panel Reactive Antibodies ≥6% (<6% is reference) (Dutch cohort) resp. Highly sensitized patient (UK cohort)	1.420 (1.095-1.840)	0.008	0.792 (0.661-0.950)	0.012

Factors removed during the backward stepwise regression analysis were: donor sex, -terminal creatinine, - history of diabetes, -cause of death; donor type; recipient age, - sex, -BMI, and HLA-A, -B and Dr mismatches.

⁎ Cases of EGL in recipients who died with a functioning graft within 90 days of transplantation were excluded. ns: not significant.

The slightly lower than expected by change alone incidences of paired EGLs, and modest performance characteristics (r^2^) of statistical prediction models that include all major donor characteristics suggest that the contribution of donor factors to EGL might be less than commonly assumed.

**Figure 2:**
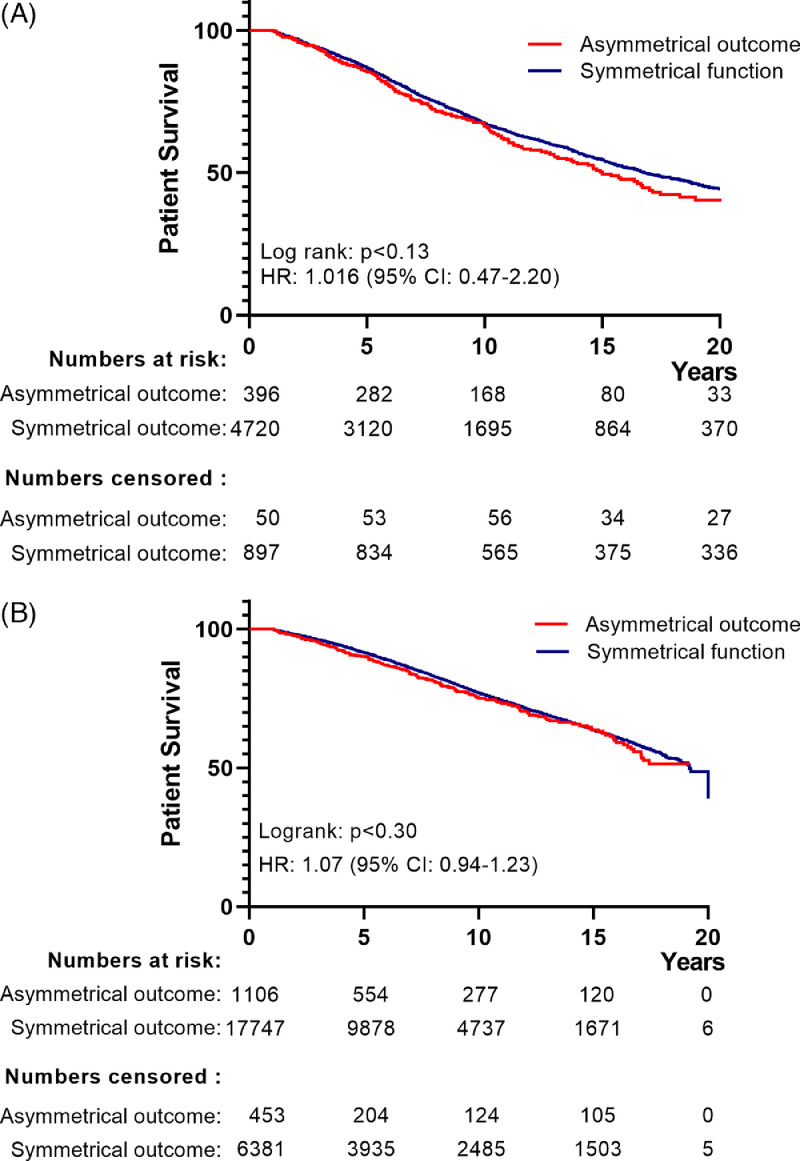
*(A) Kaplan-Meier curves of ‘patient survival following transplantation’ for Dutch recipients receiving a graft from donor pairs with symmetrical function vs. those receiving a graft from a donor pair with asymmetrical function for transplantations performed.* The blue curve represents those receiving a graft from pairs with symmetrical function, the red curve represents recipients of a graft from pairs in which the contralateral graft was lost because of EGL_(censored for death with a functioning graft)_. The accompanying table shows the number of events along with the number of patients at risk and censored over time. Data censored for recipients dying within 90-days of transplantation (n=148). A log-rank test against the hypothesis of equal hazard rates gives p-value of 0.13. *(B) Kaplan-Meier curves of ‘patient survival following transplantation’ for UK recipients receiving a graft from donor pairs with symmetrical function vs. those receiving a graft from a donor pair with asymmetrical function for transplantations performed.* The blue curve represents those receiving a graft from pairs with symmetrical function, the red curve represents recipients of a graft from pairs in which the contralateral graft was lost because of EGL_(censored for death with a functioning graft)_. The accompanying table shows the number of events along with the number of patients at risk and censored over time. Data censored for recipients dying within 90-days of transplantation (n=211). A log-rank test against the hypothesis of equal hazard rates gives p-value of 0.30.

**Figure 3:**
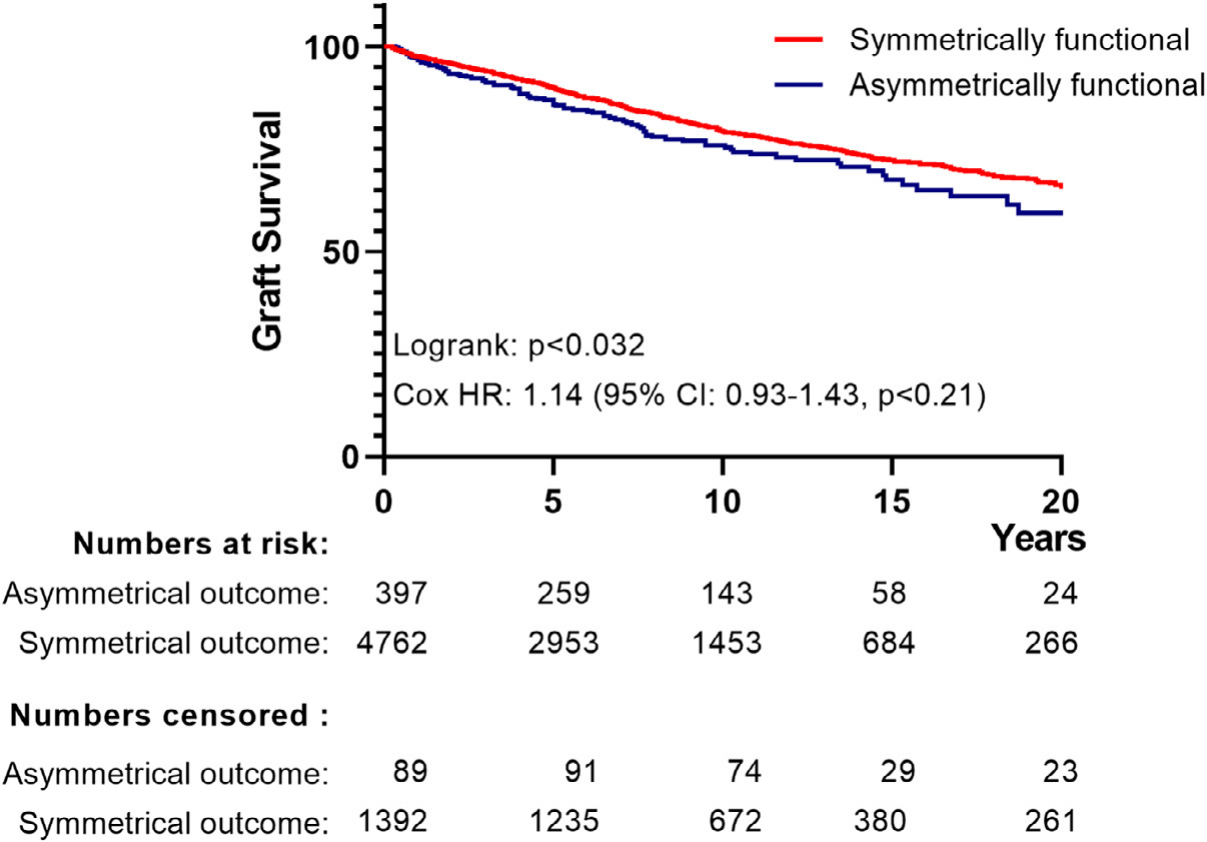
*Kaplan–Meier curves of graft survival for transplantations performed in the Netherlands.* The blue curve represents grafts from pairs with symmetrical function, the red curve represents grafts from pairs in which the contralateral graft was lost because of EGL_(censored for death with a functioning graft)_. The lower table shows the number of events along with the number of patients at risk and censored over time. A log-rank test against the hypothesis of equal hazard rates gives p-value of 0.032. Significance was lost following adjustment for transplant year, and donor/recipient age (Cox regression).

**Figure 4:**
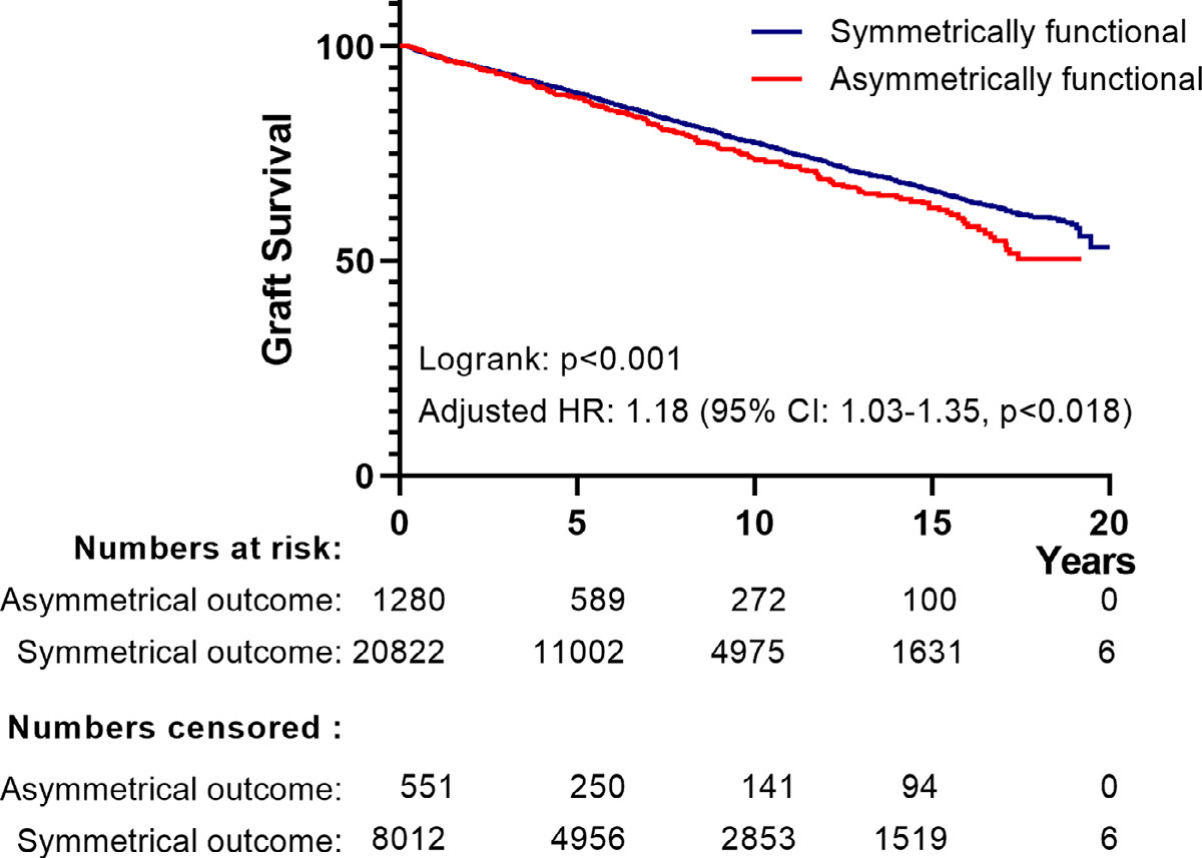
*Kaplan–Meier curves of graft survival for transplantations performed in the UK.* The blue curve represents grafts from donor pairs with symmetrical function, the red curve represents grafts from pairs in which the contralateral graft was lost because of EGL_(censored for death with a functioning graft)_. The lower table shows the number of events along with the number of patients at risk and censored over time. A log-rank test against the hypothesis of equal hazard rates gives p-value of 0.001. Adjustment for: year of transplant, donor/recipient age, HLA-A/B/Dr mismatch, and highly sensitized recipient (Cox regression analysis) resulted in an estimated p-value against the hypothesis of equal hazard rates of 0.018.

If correct, such a scenario would result in convergence of long-term outcomes for functional grafts belonging to the non-congruent outcome group (contra-lateral kidney experienced EGL_censored for death with functioning graft_), and symmetrically functional grafts (donor pairs of which neither graft experienced EGL). The Kaplan Meier curves for the Dutch and the UK cohorts (Figures 2A and B) showed similar recipient survival for the non-congruent outcome and symmetrically successful outcome groups. Evaluation of graft survival for the Dutch cohort indicated a compromised graft survival in the asymmetrical outcome group (Figure 3, logrank test: p< ·03), however this contrast was lost after correction for year-of-transplant, and donor and recipient age (HR for graft loss: 1.14 (95% CI 0.93-1.44) (Figure 3). Data for the UK cohort also indicated a compromised graft survival for the asymmetrical outcome group (logrank test: p< ·001, Figure 4). This difference persisted following correction for the year of transplantation, donor and recipient age, donor type and a diagnosis of diabetes in the donor (HR for graft loss: 1·18 [95% CI: 1.03-1·35], p< ·018).

Contrasts were observed for graft function (eGFR) with a reduced 1 and 5-years eGFR for functioning grafts from asymmetrical outcome pairs (p< ·001, Table 5), a difference that was partly reduced following correction for year of transplantation, donor and recipient age, donor type, and a history of hypertension in the donor.

**Table 5:** Uncorrected (crude) and estimated marginal (adjusted) mean 1- and 5-years functional outcomes (eGFR) for Contralateral functioning grafts, and Symmetrically no-EGL (Reference) grafts.

	Crude means (sd)		Adjusted means [95% CI]*	
	Contralateral funct.	Symm. no EGL	Contralateral funct.	Symm. no EGL
12 months eGFR	44.7 (18.4)	49.9 (19.1)	46.1 [44.9 – 47.2]	49.9 [49.6 – 50.1]
60-months eGFR	43.7 (18.4)	48.7 (20.2)	46.0 [44.5 – 47.6]	48.6 [48.3 – 49.0]

⁎ Adjusted for transplant year, donor age, recipient age and history of hypertension in the donor (ANCOVA). P_adjusted_: 12 months: 10^−9^; P_adjusted_: 60 months: 0.001.

Conclusions of these analyses are potentially interfered by the complexity of the mechanisms underlying EGL. EGL may involve recipient factors, similarly graft quality (donor factors) that may contribute to recipient's 90-days mortality. To test the robustness of the conclusions based on an analysis using EGL_censored for death with functioning graft_ as the selection criterium for the asymmetrical outcome group in the functional outcome analysis, three sensitivity analyses were performed on the UK registry data (supplemental Tables 1-3, supplemental Figures 2A-C).

A first sensitivity analysis included all EGL's (i.e. also including contralateral EGL related to death with a functioning graft). The second analysis only included EGLs caused by primary non-function, vascular thrombosis or operative problems (i.e. largely excluding immunologic aspects), and a third analysis (survival only) exclusively included contralateral EGL caused by primary non-function. Conclusions from the first and second sensitivity analyses (resp. all EGLs or EGL exclusively related to primary non-function, vascular thrombosis or operative problems) showed compromised graft survival in the asymmetrical outcome group (p< ·006, supplemental Figures 2&3). This difference that persisted following correction for the year of transplantation, donor and recipient age, donor type and a history of diabetes in the donor (HR_all EGLs_: 1·17 (95% CI: 1·04-1·33, p< ·012. No survival differences were observed in the second and third sensitivity analyses (contralateral EGL_exclusively related to primary non-function, vascular thrombosis or operative problems_ or contralateral EGL_exclusively related to primary non-function_), p< ·13 resp ·25, supplemental Figures 2B respectively 2C).

Conclusions with respect to graft function (eGFR) were minimally influenced by the sensitivity analyses, or by the statistical strategy chosen (ANCOVA or linear regression, Table 5, supplemental Tables 4A-C). Statistical significance was lost for the corrected difference in 5-years eGFR in the second sensitivity analysis (exclusively EGL related to primary non-function, vascular thrombosis or operative problems). Because of the small group size, no third functional sensitivity analysis was performed (exclusively contralateral EGL related to primary non-function).

## Discussion

This study aimed to estimate whether implementation of donor risk indices in the decision process whether not to accept a transplantable organ is justified. The study is based on data from two countries that have not implemented donor-characteristics-based decision algorithms. Initially, a large Dutch cohort followed for a prolonged period of years was analyzed to assess the reported impact of donor factors on transplant outcomes using EGL as an unequivocal transplant outcome. To enhance the robustness of the findings a similar cohort was analyzed in the UK using identical methodology. The combined analyses did show a clear role for donor-associated factors in a subgroup of early graft losses. Nevertheless, the major conclusion of the study is that, once the decision to accept the donor kidney for transplantation has been taken, donor factors minimally influence short and long-term outcomes for most transplants. Implementation of donor risk indices will result in an unjustified discard of suitable organs.

A failed transplantation or premature graft loss is a medical catastrophe with far reaching consequences.^13^^,^ As a consequence, grafts with an anticipated high risk of failure or premature loss are declined for transplantation. In many countries, the decision to accept a donor kidney offer is based on some form of donor risk estimation. Although these considerations may reduce the risk of a failed transplant and may improve overall transplant outcomes, depending on their specificity they will inevitably result in discard of potentially suitable grafts,^14^^,^^15^ thereby increasing waiting-time and wait-list mortality.

A limitation of the current policy is that the decision to accept or decline an organ is essentially based on donor characteristics, ignoring a potential impact of procedural and recipient factors on outcomes.^16^^,^^17^ Indications for a prominent role for recipient factors in transplant outcomes comes from the consistently poor performance characteristics of donor-based risk prediction models. In fact, it has been pointed out that the Kidney Donor Risk Index (KDRI), as the most widely applied donor risk index is not superior to donor age in predicting outcomes for kidney-only procedures performed in British Columbia.^18^ Similarly, registry data for the Netherlands imply an estimated (Nagelkerke's) r^2^ of 0.03 for the KDRI.^11^ This means that the variables included in the KDRI only account for approximately 3% of the variability in 5-year transplant outcomes.

It could be argued that these conclusions are interfered by differences between the donor populations in the USA, British Columbia and The Netherlands, as e.g. in the latter donors are older, there are more DCDs, and the prevalence of hepatitis C is minimal. Therefore, it is important to point out that the reported performance characteristics (c-statistics) for the optimized KDRI (c-statistics: 0.652),^5^ fully align with those for the Dutch donor population (0.63),^7^ and the estimated r^2^ of 3% indicated by the Dutch registry data^11^ aligns well with the estimated r^2^ values for a c-statistic of 0.65 (between 3-7%).^19^ Hence, the KDRI (and key donor characteristics) explain 3-7% of the variation in 5-years transplantation outcomes. This observation implies that the primary focus on donor parameters in decision-processes whether to accept or decline an organ might be less appropriate than it is perceived. This aspect would be best addressed in a prospective trial with all offered grafts being transplanted. However, such a trial will obviously be considered unethical.

Therefore, we decided for an alternative, indirect approach, and performed this instrumental variable analysis based on the outcomes of donor kidney pairs with grafts transplanted in different recipients. We reasoned that a prominent role of donor-associated factors would result in a high concordance of transplant outcomes, whereas a low concordance would be consistent with a more diffuse model. Although it could be argued that EGL is of limited clinical relevance given that is it both infrequent and notoriously difficult to predict, we chose EGL as it constitutes the most unequivocal outcome measure for this conceptual study. Longer-term outcomes such as graft survival and/or eGFR are particularly prone to interference by confounders, such as recurrent disease, differences in surveillance, and (adherence to) immune suppressive therapy. On the same token, clinically applied definitions for the short-term outcome measure -delayed graft function- are diverse, and its diagnosis is interfered by timing of the dialysis preceding transplantation, fluid overload and/or hyperpotassaemia/hyperphosphataemia, as well as by contrasting impacts in DBD and DCD donor grafts.^20^

With respect to an outcome comparison, we thought two aspects to be relevant. First, the degree of symmetry in incident EGL for donor pairs. It was reasoned that a high concordance (“symmetry”) of EGL would be consistent with a prominent role for donor factors in this outcome. The second aspect considered relevant was a comparison of transplantation outcomes for procedures with asymmetrical outcomes (i.e. grafts from a donor pair from which the other graft was lost due to EGL), with the outcomes for symmetrically successful transplantations (i.e. both grafts from a donor pair were successfully transplanted in two recipients). It was reasoned that prominent involvement of donor factors would result in segregation of transplantation outcomes with superior outcomes in symmetrically functional grafts, whereas a more discrete impact of donor factors would translate in more congruent outcomes.

Registry data for both The Netherlands and the UK indicated a slightly lower incidence of symmetrical EGL than would be predicted on basis of chance alone, and it was concluded that for the vast majority of donor pairs (>85%) EGL was asymmetrical. Consequently, these data support a more granular diffuse etiology for EGL, with a limited impact of donor factors for grafts that were both accepted for transplantation.

A limited contribution of donor factors is also reflected by the moderate differences in adverse donor factors for kidney pairs with symmetrical EGL, hence no specific donor factor was found to be dominantly associated with co-incident EGL. Similarly mapping of procedural and recipient characteristics associated with EGL in the asymmetrical outcome group indicated a relative (granular) enrichment of adverse factors for procedures in the EGL arm, but no specific procedural or recipient factor was found to prominently associate with co-incident EGL. Therefore, once the decision to accept a donor kidney pair for transplantation has been taken and the grafts have been transplanted, the impact of donor factors on early graft failure appears to be very limited.^21^

The high incidence of asymmetrical EGLs allowed for a second, alternative strategy to test the hypothesis that, once grafts are accepted for donation, donor factors have a limited impact on transplant outcomes. In this second strategy we compared the long-term outcomes of functional grafts from donor pairs with asymmetrical outcomes (i.e. pairs from which one graft was lost because of EGL) with outcomes of symmetrically successful transplantations.

This analysis showed similar graft survival for the Dutch cohort, and a slightly compromised graft survival for grafts in the asymmetrical outcome group in the UK data. Because incident EGL also involves recipient-related factors, and because donor-factors may contribute to death-with-a-functioning graft, we performed a number of sensitivity analyses to evaluate the robustness of our findings. Although the conclusions of the sensitivity analyses are to some extend inferred by reductions in sample size, conclusions from the sensitivity analysis fully aligned with the primary analysis, and even suggested that the primary analysis overestimated the potential impact of donor factors.

Functional data (1- and 5-years eGFR) for the UK cohort also allowed for a further, functional analysis. This analysis indicated a 5-10% lower eGFR in the asymmetrical outcome group. This lower eGFR did not impact recipient survival, but may (in part) contribute to the poorer graft survival in the asymmetrical outcome group (HR for graft loss 1.18, which translates in a reduction in 10-year graft survival from approx. 78% to approx. 74% (95% CI: 70.3-77·3%) Consequently, results from this second evaluation follow the conclusions for short-term outcomes, and indicate a limited role of donor factors on long-term outcomes of transplanted donor kidney pairs.

It is thus concluded that, once donor graft have been accepted for transplantation, donor characteristics will minimally impact transplant outcomes. The strong focus on donor characteristics or donor risk indices in the decision process whether or not to accept a transplantable kidney graft could result in an unjustified increase of discarded viable donor kidneys^22^^,^^23^ and avoidable deaths on the waiting list.

Our study has some limitations as this is a registry-based study on outcome evaluation of donated, and transplanted kidney pairs. This strategy obviously results in a degree of bias and confounding since the analysis is restricted to the registered variables, information for kidney pairs not accepted for transplantation is missing, and as it excludes kidneys from a single donor from whom one kidney was declined for transplantation. Moreover, although the NOTR and NTxD registries are mandatory registry for all transplant centers and several quality checks are performed, missing data and registration errors remain an issue. For example, in the UK registry a quarter of the EGL cases the underlying cause was not coded. This may result in an incomplete inclusion in the sensitivity analysis applying a more restricted definition of EGL. A second limitation is that it is exclusively based on transplanted donor kidney pairs. It is well conceivable that this results in a bias towards more favorable donor characteristics.

Also, the vast majority of the patients in this evaluation are Caucasian. Given the potential impact of race on transplant outcomes,^24^^,^^25^ any conclusion may not fully apply to non-Caucasians. Data for the asymmetrical outcome group imply a higher failure risk for right kidneys, an aspect which may relate to differences in the arterial anatomy. This phenomenon is particularly noticeable in the second sensitivity analysis using a more restricted definition of EGL (exclusively EGL caused by PNF; vascular/urethric operative problems, or vascular-thrombosis-related).

Finally, conclusions in this study are potentially influenced by confounders such as time-effects with improved outcomes over time, and by medical decision-making such as the introduction of the old-for-old transplantation program in the Netherlands and the longevity matching in the UK.

In conclusion, this study based on the data of two national registries suggests that, once the decision to accept kidneys from a deceased donor for transplantation has been taken, outcomes for most transplants reflect a complex interplay of donor-, procedural- and, in particular recipient factors. Efforts to optimize transplant outcomes should focus on a better understanding of the recipient factors underlying transplant outcomes.

## Contributors

AFS: conceptualization, data interpretation, drafting manuscript. MK: Data acquisition, reviewing. LM: Data acquisition, reviewing. MR: Data acquisition, reviewing. RJ: Data acquisition, reviewing. MK: Data analysis, reviewing . FJB: Data acquisition, reviewing . JvdW: Data acquisition, reviewing. ADvZ: Data acquisition, reviewing. MHLC: Data acquisition, reviewing. MCB: Data acquisition, reviewing. ASN: Data acquisition, reviewing. SPB: Data acquisition, reviewing. EB: Data interpretation, reviewing. APJdV: Data acquisition, reviewing. ES: Data analysis, data interpretation, reviewing. RJP: Data acquisition, data interpretation, reviewing. JHNL: Data analysis, data interpretation, drafting manuscript.

## Data sharing statement

This study is based on data made available by the Netherlands Organ.

Transplant Registry (NOTR) and the UK National Transplant Database (NTxD). Analyses can be available upon reasonable request to the corresponding author.

## Funding

None.

## Declaration of interests

The authors have nothing to disclose.

## Appendix Supplementary materials

Image, application 1
