# Supplementary material for: Donor characteristics and their impact on kidney transplantation outcomes: Results from two nationwide instrumental variable analyses based on outcomes of donor kidney pairs accepted for transplantation
Source: eClinicalMedicine. 2022 Jun 25;50:101516. doi: 10.1016/j.eclinm.2022.101516 (PMC9240982; doi:10.1016/j.eclinm.2022.101516)

**SUPPLEMENTAL TABLE 1A. Donor characteristics of cases included in sensitivity analyses incorporating all cases (i.e. no censoring for early deaths with a functioning graft for all 23,900 paired kidney transplantations performed in the UK between 2000 and 2018).** Mean (sd) or Median [IQR].

|  | **Double sided EGL**  **n=208^1^**  **104 donor pairs** | **Asymmetrical EGL**  **n=2928**  **1464 donor pairs** | **Symmetrical no EGL**  **“Reference”**  **n=20760**  **10780 donor pairs** |
| --- | --- | --- | --- |
| **DCD (%)** | 40.4 | 31.1 | 31.5 |
| **Sex donor (male) (%)** | 61.5 | 53.3 | 53.6 |
| **Age donor (yr)**  **mean (sd)**  **median [IQR]** | 56.5 (12.0)  58.5 [50.0-66.0] | 52.7 (13.5)  54.0 [45.0-63.0] | 49.9 (15.0)  52.0 [41.0-60.0] |
| **% Donors >60 yrs** | 49.0 | 33.0 | 27.2 |
| **Body-mass index donor (kg/m^2^)** | 26.5 (6.1) | 27.1 (5.2) | 27.1 (5.2) |
| **Last creatinine donor (μmol/l)**  **Mean (sd)**  **Median IQR** | 96 (61)  85 [61-116] | 86 (45)  74 [60 – 97] | 85 (45)  74 [58-96] |
| **eGFR (MDRD) donor (ml/min)**  **Median [IQR]** | 80.5 [54.3-116.0] | 84.1 [63.5-107.3] | 85.9 [64.3-110.6] |
| **Cause of death donor (%)**  **Trauma**  **Stroke**  **Cardiac arrest**  **Other** | 21.8  64.4  2.0  11.9 | 21.4  69.3  0.9  8.2 | 27.8  62.3  1.1  8.8 |
| **Expanded criteria donor (%)** | 59.6 | 43.7 | 35.2 |
| **History of diabetes (%)** | 5.9 | 7.2 | 6.0 |
| **History of hypertension (%)** | 38.5 | 32.1 | 25.8 |
| **History of smoking (%)** | 45.0 | 52.7 | 51.1 |

**SUPPLEMENTAL TABLE 1B. Procedural and recipient characteristics of cases included in sensitivity analyses incorporating all cases (i.e. no censoring for early deaths with a functioning graft for all 23,900 paired kidney transplantations performed in the UK between 2000 and 2018).** Mean (sd) or Median [IQR].

|  | **Double sided EGL**  **n=208**  **104 donor pairs** | **Asymmetrical EGL**  **n=1466** | **Contralateral functioning graft**  **n=1466** | **Symmetrical no EGL**  **“Reference”**  **n=20760**  **10780 donor pairs** |
| --- | --- | --- | --- | --- |
| **Left kidney (%)** | NA | 45.8 | 54.2 | NA |
| **First Warm ischemia time (min.), (DCD only)**  **Mean (sd)**  **Median [IQR]** | 22.8 (8.8)  19.0 [14.8-32.0] | 26.1 (27.8)  18.0 [15.0-25.0] | 23.3 (18.0)  18.0 [15.0 – 24.0] | 22.6 (20.5)  18.0 [15.0-23.0] |
| **Cold ischemia time (hrs)**  **Cold ischemia time**  **distribution (%)**  **<12 hrs**  **12-18 hrs**  **18-24 hrs**  **>24 hrs** | 16.4 (6.1)  30.8  40.9  20.7  7.7 | 17.1 (6.3)  23.9  40.3  24.8  10.9 | 16.4 (6.1)  27.0  41.9  22.2  8.9 | 16.2 (5.9)  24.5  43.9  22.2  9.4 |
| **Graft anastomosis time (min)**  **Median [IQR]** | 12.8 (4.1)  12.0 [10.0 – 15.0] | 19.3 (28.5)  13.0 [10.0-16.0] | 16.3 (20.2)  13.0 [11.0 – 16.0] | 15.5 (19.5)  13.0 [10.0 – 15.0] |
| **KDRI** | 1.12 (0.38) | 1.08 (0.25) | 1.07 (0.25) | 1.08 (0.37) |
| **DGF (%)** | NA | NA | 24.6 | 21.3 |
| **Sex recipient (male) (%)** | 60.6 | 62.0 | 63.5 | 63.0 |
| **Age recipient (years)**  **Median [IQR]** | 53.0 (11.9)  54.0 [44.0-62.3] | 52.1 (13.2)  53.0 [43.0-62.0] | 51.3 (13.1)  53.0 [42.0 – 61.0] | 50.2 (13.3)  51.0 [41.0 – 60.0] |
| **BMI recipient (kg/m²)** | 27.7 (4.5) | 27.1 (4.9) | 26.6 (4.8) | 26.5 (4.8) |
| **Diabetes** | 8.6 | 10.0 | 7.5 | 8.5 |
| **Wait time (years, median (IQR))** | 2.5 [1.3 – 4.2] | 2.5 [1.1 – 4.2] | 2.1 [0.9 – 3.8] | 2.1 (0.9 - 3.7) |
| **Previous transplants**  **0**  **1**  **2**  **more** | 83.2  14.4  1.9  0.4 | 82.3  14.1  3.3  0.3 | 75.4  11.3  1.7  0.1 | 85.2  12.4  2.1  0.1 |
| **mismatches (%)**  **HLA-Dr 0**  **1**  **2**  **HLA-A 0**  **1**  **2**  **HLA-B 0**  **1**  **2** | 50.9  42.8  6.3  19.7  55.3  25.0  14.4  72.6  13.0 | 52.6  43.9  3.5  22.0  49.0  28.9  17.4  68.7  13.9 | 55.9  40.1  4.0  23.7  51.5  24.8  19.2  66.7  14.1 | 57.0  39.5  3.5  23.2  50.7  26.1  19.2  67.9  12.9 |
| **Highly immunized patient** | 10.6 | 9.7 | 7.7 | 8.3 |
| **Induction therapy**  **Anti-IL2r**  **ATG** | 69.3  3.9 | 68.8  4.5 | 69.3  4.3 | 72.7  3.4 |
| **Initial immune suppression (%)**  **Azothioprine**  **Ciclosporin A**  **Tacrolimus**  **Mycophenolate**  **Corticosteroids** | 16.1  24.3  71.2  64.9  88.3 | 15.3  23.2  71.7  68.7  85.6 | 16.8  22.1  76.2  66.2  85.6 | 15.9  19.6  78.7  68.8  84.4 |
| **Cause of early graft loss %**  **Rejection**  **Primary non function**  **Thrombosis/infarction**  **Technical**  **Infection**  **Recurrent disease**  **Death with function graft**  **Other**  **Non coded** | 11.4  12.4  15.8  12.4  1.9  1.0  9.0  13.8  19.0 | 8.5  11.4  12.7  12.8  0.8  0.6  12.9  13.0  25.5 |  |  |
| **12 months eGFR** | n.a. | n.a. | 45.0 (18.5)   1. [32.0 – 55.5] | 49.9 (19.1)  48.0 [36.0 – 61.0] |

**SUPPLEMENTAL TABLE 2A. Donor characteristics of cases included in sensitivity analysis 2 for the long-term outcomes of symmetrically functional, and asymmetrical outcome pairs (Exclusively EGL caused by PNF; vascular/urethric operative problems, or vascular-thrombosis for all 23,900 paired kidney transplantations performed in the UK between 2000 and 2018).** Mean (sd) or Median [IQR].

|  | **Double sided EGL**  **n=48** | **Asymmetrical EGL**  **n=594 (EGL)** | **Symmetrical no EGL**  **“Reference”**  **n=17558**  **8779 donor pairs** |
| --- | --- | --- | --- |
| **DCD (%)** | 41.7 | 31.6 | 31.5 |
| **Sex donor (male) (%)** | 56.3 | 51.5 | 53.6 |
| **Age donor (yr) mean (sd)**  **median [IQR]** | 56.3 (13.9)  61.0 [59.3- 66.0] | 52.5 (13.2)  54.0 [45.0-62.0] | 49.9 (15.0)  52.0 [41.0-60.0] |
| **Body-mass index donor (kg/m^2^)** | 25.9 (5.2) | 27.3 (5.6) | 27.1 (5.4) |
| **Last creatinine donor (μmol/l)** | 96 (45)  88 [67 – 116] | 84 (37)  75 [61 – 97] | 86 (51)  74 [58-96] |
| **eGFR (MDRD) donor (ml/min)** | 80.5 [54.3-116.0] | 84.1 [63.5-107.3] | 85.9 [64.3-110.6] |
| **Cause of death donor (%)**  **Trauma**  **Stroke**  **Cardiac arrest**  **Other** | 16.7  79.2  4.2  0 | 21.0  69.7  1.7  7.6 | 27.8  62.3  1.1  8.8 |
| **Expanded criteria donor (%)** | 60.4 | 43.1 | 35.9 |
| **History of diabetes (%)** | 0 | 6.1 | 6.0 |
| **History of hypertension (%)** | 26.2 | 32.2 | 25.8 |
| **History of smoking (%)** | 43.2 | 54.0 | 51.1 |

^1^

**SUPPLEMENTAL TABLE 2B. Procedural and recipient characteristics of cases included in sensitivity analysis 2 for the long-term outcomes of symmetrically functional, and asymmetrical outcome pairs. (Exclusively EGL caused by PNF; vascular/urethric operative problems, or vascular-thrombosis for all 23,900 paired kidney transplantations performed in the UK between 2000 and 2018).** Mean (sd) or Median [IQR].

|  | **Double sided EGL**  **n=48** | **EGL**  **n=594** | **Contralateral functioning graft**  **n=551** | **Symmetrical no EGL**  **“Reference”**  **n=17558**  **8779 donor pairs** |
| --- | --- | --- | --- | --- |
| **Left kidney (%)** | 50% | 38.7 | 61.5 | 50% |
| **First warm ischemia time (min.) (DCD only)**  **Median [IQR]** | 14.3 (2.3)^1^ | 28.2 (26.9)  20.0 [15.3-28.8] | 28.7 (29.2)  20.0 [15.0 – 27.5] | 22.6 (20.5)  18.0 [15.0-23.0] |
| **Cold ischemia time (hrs)**  **Cold ischemia time**  **distribution (%)**  **<12 hrs**  **12-18 hrs**  **18-24 hrs**  **>24 hrs** | 17.7 (7.3)  30.8  40.9  20.7  7.7 | 17.8 (6.5)  23.9  40.3  24.8  10.9 | 16.7 (5.8)  27.0  41.9  22.2  8.9 | 16.2 (5.9)  24.5  43.9  22.2  9.4 |
| **Graft anastomosis time (min)**  **Median [IQR]** | 11.7 (3.1)  13.0 [9.0-14.0] | 17.6 (24.1)  13.0 [10.5-15.5] | 18.4 (25.1)  13.0 [11.0 – 16.0] | 15.5 (19.5)  13.0 [10.0 – 15.0] |
| **KDRI** | 1.12 (0.38) | 1.08 (0.25) | 1.07 (0.25) | 1.08 (0.37) |
| **DGF (%)** | NA | NA | 33.3 | 27.9 |
| **Sex recipient (male) %** | 64.6 | 62.6 | 63.5 | 63.0 |
| **Age recipient (years)** | 51.6 (12.0)  51.0 [42.0-61.0] | 50.6 (13.1)  51.0 [45.0-62.5] | 50.8 (12.7)  52.0 [41.0 – 61.0] | 50.2 (13.3)  51.0 [41.0 – 60.0] |
| **BMI recipient (kg/m²)** | 27.6 (3.5) | 27.2 (4.8) | 26.0 (4.6) | 26.5 (4.8) |
| **Diabetes %** | 12.5 | 8.4 | 8.0 | 8.5 |
| **Wait time (years, median (IQR))** | 2.1 [1.2 – 3.8] | 2.3 (1.1 – 4.0) | 2.1 (0.8 - 3.9) | 2.1 [0.9 - 3.7] |
| **Previous transplants**  **0**  **1**  **2**  **more** | 87.5  10.4  2.1  0.0 | 81.6  14.4  3.9  0.0 | 85.7  12.7  1.5  0.0 | 85.2  12.9  2.1  0.3 |
| **mismatches (%)**  **HLA-Dr 0**  **1**  **2**  **HLA-A 0**  **1**  **2**  **HLA-B 0**  **1**  **2** | 41.7  58.3  6.3  22.9  58.3  18.8  16.7  77.1  6.3 | 52.4  43.4  4.2  23.9  50.5  27.6  17.8  68.0  14.1 | 56.3  39.4  4.4  26.9  48.6  24.5  22.0  64.8  13.2 | 57.0  39.5  3.5  23.2  50.7  26.1  19.2  67.9  12.9 |
| **Highly immunized patient** | 8.3 | 8.4 | 8.1 | 8.3 |
| **Induction therapy**  **Anti-IL2r**  **ATG** | 59.6  2.1 | 65.2  5.1 | 62.8  3.7 | 72.7  3.4 |
| **Initial immune suppression (%)**  **Azothioprine**  **Ciclosporin A**  **Tacrolimus**  **Mycophenolate**  **Corticosteroids** | 21.3  31.3  68.1  66.0  97.9 | 17.3  28.1  65.9  65.2  81.2 | 20.5  26.5  72.2  62.5  83.7 | 15.9  19.6  78.7  68.8  84.4 |
| **Cause of early graft loss %**  **Rejection**  **Primary non function**  **Thrombosis/infarction**  **Technical**  **Infection**  **Recurrent disease**  **Other** | 11.7  19.2  22.5  18.3  1.7  1.7  25.0 | 9.6  15.4  22.9  21.6  1.5  0.7  21.7 |  |  |
| **12 months eGFR** | n.a. | n.a. | 43.5 (17.5)  42.0 [31.0 -55.0] | 49.9 (19.1)  48.0 [36.0 – 61.0] |

**SUPPLEMENTAL TABLE 3A. Donor characteristics of cases included in sensitivity analysis 3 for the long-term outcomes of symmetrically functional, and asymmetrical outcome pairs (Exclusively EGL caused by PNF, for all 23,900 paired kidney transplantations performed in the UK between 2000 and 2018).** Mean (sd) or Median [IQR].

|  | **Double sided EGL**  **n=34** | **Asymmetrical EGL**  **n=167 (EGL)** | **Symmetrical no EGL**  **“Reference”**  **n=17558**  **8779 donor pairs** |
| --- | --- | --- | --- |
| **DCD (%)** | 47.1 | 39.5 | 31.5 |
| **Sex donor (male) (%)** | 70.6 | 53.9 | 53.6 |
| **Age donor (yr) mean (sd)**  **median [IQR]** | 59.2 (14.1)  64.0 [53.8-68.0] | 54.4 (13.2)  56.0 [48.0-63.0] | 49.9 (15.0)  52.0 [41.0-60.0] |
| **Body-mass index donor (kg/m^2^)** | 26.9 (6.2) | 26.4 (5.2) | 27.1 (5.4) |
| **Last creatinine donor (μmol/l)** | 151 (123)  95 [86-172] | 88 (46)  77 [61-100] | 86 (51)  74 [58-96] |
| **eGFR (MDRD) donor (ml/min)** |  |  | 85.9 [64.3-110.6] |
| **Cause of death donor (%)**  **Trauma**  **Stroke**  **Cardiac arrest**  **Other** | 23.5  58.8  11.8  5.9 | 19.2  70.7  0  10.2 | 27.8  62.3  1.1  8.8 |
| **Expanded criteria donor (%)** | 64.7 | 46.1 | 35.9 |
| **History of diabetes (%)** | 5.8 | 9.4 | 6.0 |
| **History of hypertension (%)** | 43.8 | 33.5 | 25.8 |
| **History of smoking (%)** | 50.0 | 55.0 | 51.1 |

^1^

**SUPPLEMENTAL TABLE 3B. Procedural and recipient characteristics of cases included in sensitivity analysis 3 for the long-term outcomes of symmetrically functional, and asymmetrical outcome pairs (Exclusively EGL caused by PNF, for all 23,900 paired kidney transplantations performed in the UK between 2000 and 2018).** Mean (sd) or Median [IQR].

|  | **Double sided EGL**  **n=48** | **EGL**  **n=594** | **Contralateral functioning graft**  **n=551** | **Symmetrical no EGL**  **“Reference”**  **n=17558**  **8779 donor pairs** |
| --- | --- | --- | --- | --- |
| **Left kidney (%)** | 50 | 50.6 | 49.4 | 50 |
| **First warm ischemia time (min.) (DCD only)**  **Median [IQR]** | 34 [21-37]  30.5 (9.4) | 21 [15-24]  21.5 (9.0) | 20 [15-25]  26.6 (28.7) | 22.6 (20.5)  18.0 [15.0-23.0] |
| **Cold ischemia time (hrs)**  **Cold ischemia time**  **distribution (%)**  **<12 hrs**  **12-18 hrs**  **18-24 hrs**  **>24 hrs** | 15.6 (6.2)  25.0  50.0  12.5  12.5 | 18.2 (6.7)  12.3  44.4  27.8  15.4 | 16.9(6.1)  18.4  45.4  25.8  10.4 | 16.2 (5.9)  24.5  43.9  22.2  9.4 |
| **Graft anastomosis time (min)**  **Median [IQR]** | 10.8 (7.8)  14 [10-17] | 12.2 (5.4)  12 [10-16] | 18.0 (26.8)  12 [11-17] | 15.5 (19.5)  13.0 [10.0 – 15.0] |
| **DGF (%)** | NA | NA | 41.1 | 27.9 |
| **Sex recipient (male) %** | 58.9 | 65.9 | 61.3 | 63.0 |
| **Age recipient (years)** | 52.0 [42.3-64.0] | 55.0 [44.0-64.0] | 53.0 [42.0-61.0] | 50.2 (13.3)  51.0 [41.0 – 60.0] |
| **BMI recipient (kg/m²)** | 29.1 (3.9) | 27.2 (4.8) | 26.4 (4.0) | 26.5 (4.8) |
| **Diabetes %** | 8.8 | 9.0 | 6.7 | 8.5 |
| **Wait time (years, median (IQR))** | 2.7 [1.7-5.3] | 2.9 [1.6-4.7] | 2.5 [0.9-4.0] | 2.1 [0.9 - 3.7] |
| **Previous transplants**  **0**  **1**  **2**  **more** | 85.3  11.8  2.9  0 | 82.0  14.4  3.6  0 | 93.3  5.5  1.2  0 | 85.2  12.9  2.1  0.3 |
| **mismatches (%)**  **HLA-Dr 0**  **1**  **2**  **HLA-A 0**  **1**  **2**  **HLA-B 0**  **1**  **2** | 38.2  52.9  8.8  20.6  47.1  32.4  17.6  73.5  8.8 | 46.7  49.1  4.2  23.4  52.7  24.0  15.0  65.3  19.8 | 55.2  39.3  5.5  23.9  47.9  28.2  17.8  65.0  17.2 | 57.0  39.5  3.5  23.2  50.7  26.1  19.2  67.9  12.9 |
| **Highly immunized patient** | 14.7 | 10.8 | 6.8 | 8.3 |
| **Induction therapy**  **Anti-IL2r**  **ATG** | 73.6  0 | 75.5  6.2 | 69.9  3.1 | 72.7  3.4 |
| **Initial immune suppression (%)**  **Azothioprine**  **Ciclosporin A**  **Tacrolimus**  **Mycophenolate**  **Corticosteroids** | 20.6  23.5  73.5  61.8  97.1 | 13.6  23.5  72.2  70.4  81.6 | 20.2  20.2  79.1  62.8  82.2 | 15.9  19.6  78.7  68.8  84.4 |
| **Cause of early graft loss %**  **Primary non function** | 100 | 100 |  |  |
| **12 months eGFR** |  |  | 41.1 (17.6)  39.0 [26.0-52.0] | 49.9 (19.1)  48.0 [36.0 – 61.0] |

**Supplemental table 4A.** Uncorrected (crude) and estimated marginal (adjusted) mean 1- and 5-years functional outcomes (eGFR) for Contralateral functioning grafts, and Symmetrically no-EGL (Reference) grafts. Sensitivity analysis 1: contralateral graft EGL. No censoring for deaths with functioning graft.

|  | Crude means (sd) | | Adjusted means [95% CI]^*^ | |
| --- | --- | --- | --- | --- |
|  | Contralateral EGL | Symm. no EGL | Contralateral EGL | Symm. no EGL |
| 12 months eGFR | 45.1 (18.5) | 49.9 (19.1) | 46.8 [45.9 – 47.7] | 49.8 [49.6 – 50.0] |
| 60-months eGFR | 44.4 (19.0) | 48.7 (20.2) | 46.6 [45.2 – 47.9] | 48.5 [48.2 – 48.9] |

^*)^Adjusted for transplant year, donor age, recipient age and history of hypertension in the donor (ANCOVA).

**Supplemental table 4B.** Uncorrected (crude) and estimated marginal (adjusted) mean 1- and 5-years functional outcomes (eGFR) for Contralateral functioning grafts, and Symmetrically no-EGL (Reference) grafts. Sensitivity analysis 2: Contralateral graft PNF; vascular/ureteric operative problems, or vascular-thrombosis-related EGL.

|  | Crude means (sd) | | Adjusted means [95% CI]^*^ | |
| --- | --- | --- | --- | --- |
|  | Contralateral EGL | Symm. no EGL | Contralateral EGL | Symm. no EGL |
| 12 months eGFR | 43.5 (17.5) | 49.9 (19.1) | 45.9 [44.5 – 47.4] | 49.9 [49.6 – 50.1] |
| 60-months eGFR | 44.9 (18.7) | 48.7 (20.2) | 47.3 [45.3 – 49.3] | 48.6 [48.3 – 48.9] |

^*)^Adjusted for transplant year, donor age, recipient age and history of hypertension in the donor (ANCOVA).

**Supplemental table 4C**. Differences in eGFR (ml/min) between the asymmetrical outcome cohort (primary outcome and sensitivity analyses) and reference cohort estimated by linear regression analysis. B [95% CI].

|  | Primary Outcome | Sensitivity Analyses | | |
| --- | --- | --- | --- | --- |
|  | EGL_censored_ | Analysis 1 (EGL all) | Analysis 2 PNFext | Analysis 3. PNFonly |
| 12 months eGFR | 3.92 [2.77-5.07] | 3.12 [2.17-4.07] | 3.92 [2.42-5.42] | 0.34 [-4.59-4.86] |
| 60-months eGFR | 2.59 [1.04-4.14] | 2.03 [0.65-3.40] | 1.28 [-0.722-3.28] | 4.02 [-1.98-10.03] |

^*)^Adjusted for transplant year, donor age, recipient age and history of hypertension in the donor


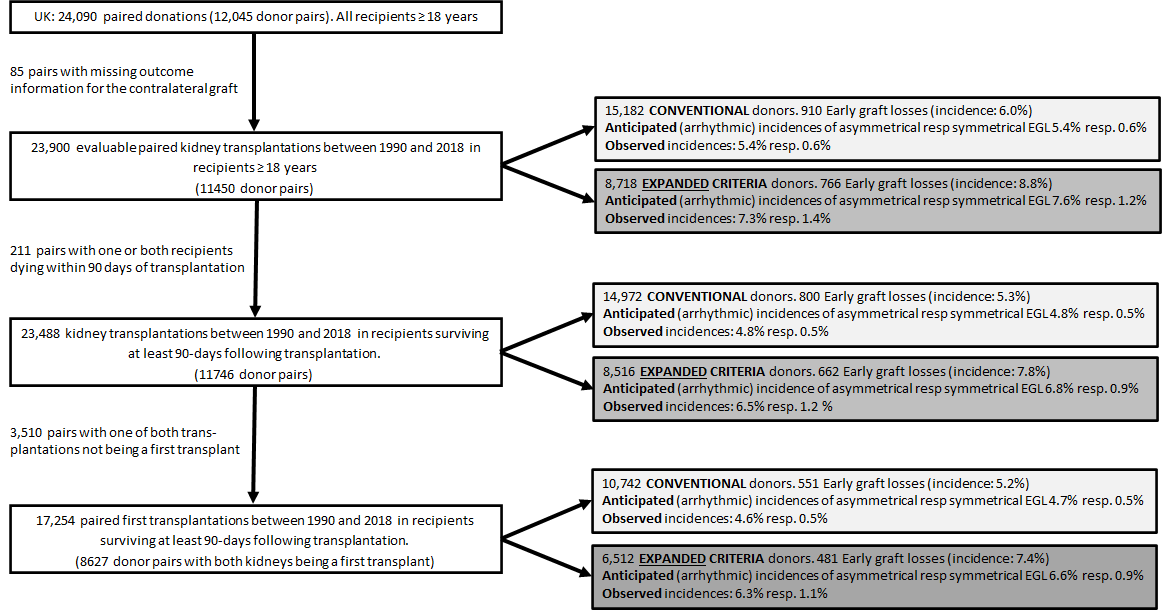
**SUPPLEMENTAL FIGURE 1 Sensitivity analysis for the distribution of observed and anticipated (arrhythmic)^*^ symmetrical (paired) and asymmetrical Early Graft Losses in the United Kingdom stratified by donor quality (i.e. conventional donors and expanded criteria donors). The primary analysis was performed for death-censored EGL (middle boxes). Two additional analyses were performed (lower and upper set of boxes): one based on all cases of EGL (not death-censored), and a second exclusively focusing on for primary transplantations.**

^*)^Anticipated (arrhythmic) distribution of symmetrical and asymmetrical EGL equals: observed incidence (%) = 2X^2^ + X (i.e. incidence of symmetrical EGL equals 2X^2^; incidence of asymmetrical EGL equals X).

**SUPPLEMENTAL FIGURE 2A.** Graft survival for grafts from donor pairs with symmetrical outcome vs. grafts asymmetrical outcome in the UK Registry data. Sensitivity analysis 1 (all EGL cases, including those related to death with a functioning grafts).

**
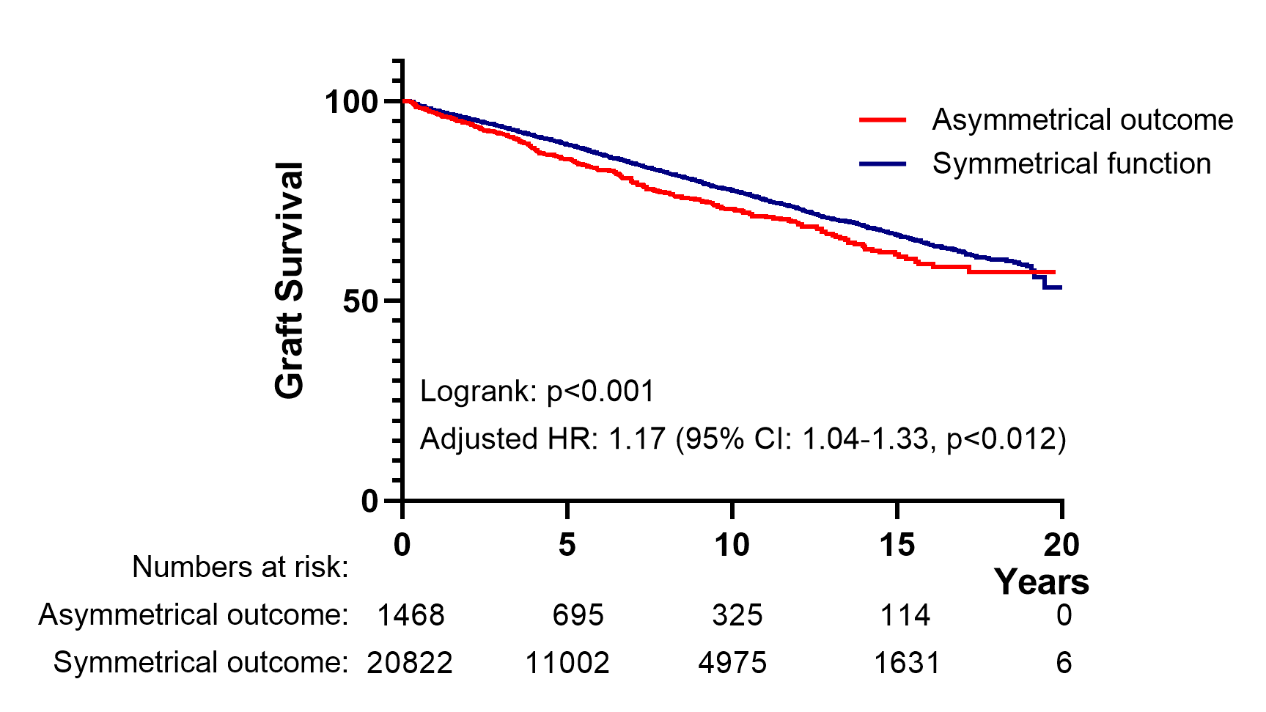
**

Cox Hazard Ratio adjusted for: year of transplant, donor/recipient age, HLA-B/Dr mismatch, and highly sensitized recipient).

**SUPPLEMENTAL FIGURE 2B.** Graft survival for grafts from donor pairs with symmetrical outcome vs. grafts asymmetrical outcome in the UK Registry data. Sensitivity analysis 2 (exclusively EGL caused by PNF; vascular/urethric operative problems, or vascular-thrombosis-related cases).

**
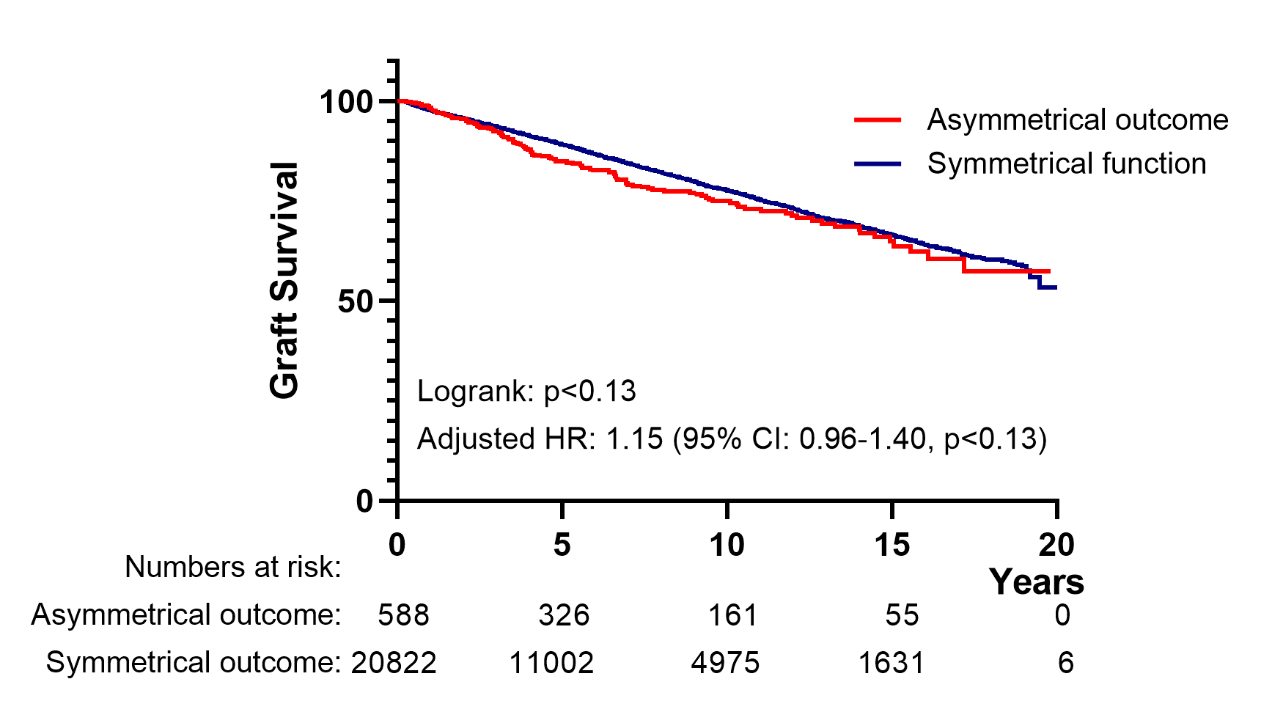
**

**SUPPLEMENTAL FIGURE 2C.** Graft survival for grafts from donor pairs with symmetrical outcome vs. grafts asymmetrical outcome in the UK Registry data. Sensitivity analysis 3 (exclusively EGL caused by PNF).


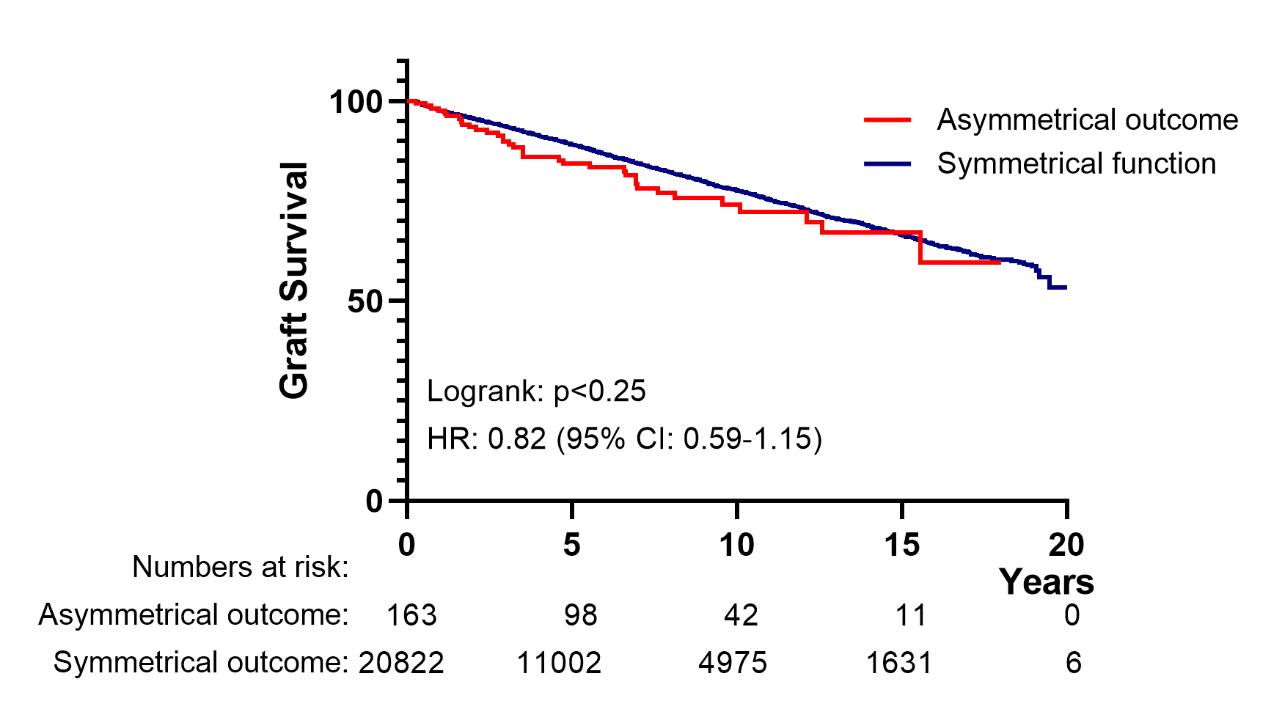

Supplement: Supplementary file 1 [file mmc1.docx]
